# Haematology specimen acceptability: a national survey in Chinese laboratories

**DOI:** 10.11613/BM.2018.030704

**Published:** 2018-10-15

**Authors:** Yuanyuan Ye, Wei Wang, Haijian Zhao, Falin He, Kun Zhong, Shuai Yuan, Yuxuan Du, Bingquan Chen, Zhiguo Wang

**Affiliations:** 1 National Center for Clinical Laboratories, Beijing Hospital, National Center of Gerontology, Beijing, China; 2 Graduate School of Peking Union Medical College, Beijing, China; 3 Beijing Clinet Information Technology Co., Ltd, Beijing, China

**Keywords:** quality indicator, preanalytical phase, quality control, haematology, survey

## Abstract

**Introduction:**

Specimen adequacy is a crucial preanalytical factor affecting accuracy and usefulness of test result. The aim of this study was to determine the frequency and reasons for rejected haematology specimens, preanalytical variables which may affect specimen quality, and consequences of rejection, and provide suggestions on monitoring quality indicators as to obtain a quality improvement.

**Materials and methods:**

A cross-sectional survey was conducted and a questionnaire was sent to 1586 laboratories. Participants were asked to provide general information about institution and practices on specimen management and record rejections and reasons for rejection from 1^st^ to 31^st^ July.

**Results:**

A total survey response rate was 56% (890/1586). Of 10,181,036 tubes received during the data collection period, 11,447 (0.11%) were rejected, and the sigma (σ) was 4.6. The main reason for unacceptable specimens was clotted specimen (57%). Rejected specimens were related to source department, container type, container material type, transportation method and phlebotomy personnel. The recollection of 84% of the rejected specimens was required. The median specimen processing delay in inpatient, outpatient and emergency department were 81.0 minutes, 57.0 minutes and 43.3 minutes, respectively.

**Conclusions:**

Overall, rejection rate was a slightly lower than previously published data. In order to achieve a better quality in the preanalytical phase, haematology laboratories in China should pay more attention on training for phlebotomy and sample transportation, identify main reasons for clotted specimen and take effective measures. The platform in the study will be helpful for long-term monitoring, but simplification and modification should be introduced in the following investigation.

## Introduction

Although laboratory medicine seems overall less vulnerable to mistakes and violations than other clinical and diagnostic areas, the chance of errors is still not negligible and may generate adverse consequences on both the quality of testing and patient safety (*1*). For errors in total testing process (TTP), preanalytical errors have been found at the majority of the total errors (46 - 68%) in laboratory medicine, mainly emerging from manually intensive activities, especially those related to collection, handling, transportation, preparation and storage of diagnostic specimens. Less errors occur in the analytical (7 - 13%) and postanalytical (18 - 47%) phase (*1*, *2*). Specimen adequacy is a crucial preanalytical factor affecting the accuracy and usefulness of test result (*3*). Laboratories usually establish a guideline for evaluating the acceptance of submitted specimens, and specimens not meeting the criteria of acceptability are rejected. Data on rejected samples due to various types of preanalytical errors are one of laboratory medicine preanalytical quality indicators (*4*).

Many agencies have developed surveys on specimen acceptability. The College of American Pathologists (CAP) Q-Probes analysis determined the frequency and reasons for rejection of chemistry specimens and complete blood count specimens and quantified the clinical consequences of specimen rejection (*3*, *5*, *6*). A retrospective analysis of the preanalytical phase had been carried out in Spain (*7*). In addition, several papers have been published concerning preanalytical errors and rejected specimens among different laboratories (*4*, *8*, *9*).

Preanalytical errors start to occur at the point of entry for laboratory test requests by clinicians (*4*). Studies showed that the main rejection reasons for haematology specimens were clotted samples and insufficient specimen quantity (*5*, *10*). Personal impact on specimen collection was an important factor and the preanalytical error rate was 2 to 4 times higher for non-laboratory phlebotomists than laboratory staff (*4*).

An investigation on specimen acceptability for complete blood count testing and coagulation testing was conducted in 2012, but it was just a general preliminary survey (*11*). In the past 5 years, more attention has been paid on preanalytical phase and laboratory information system (LIS) was continuously optimized. It called the need to know the present situation of preanalytical haematology specimen acceptability in China. The aim of this study was to determine the frequency and reasons for rejected haematology specimens, identify preanalytical variables which may affect specimen quality and consequences of rejection, and provide suggestions on monitoring quality indicators.

## Materials and methods

### Study design

A cross-sectional survey on specimen acceptability was initiated by the National Center for Clinical Laboratories (NCCL) in China and the study was conducted as part of the regular external quality assessment (EQA) scheme for 2017. Clinical laboratories participating in EQA programmes of clinical haematology were enrolled in this study, excluding non-hospital laboratories, such as commercial laboratories, centers for disease control and prevention, blood donation and supply institutions. A web-based questionnaire and survey notice were sent online on June 15^th^ 2017, along with that, a short message was sent to laboratory directors explaining the purpose of the study. Participants were asked to give a response before 31^th^ August and all data were collected *via* special software designed by NCCL and developed by CLInet Information Technology (Beijing, China).

### Survey

The questionnaire was divided into two parts. The first part contained 18 questions on institution characteristics and laboratory practices on collection, transportation, and specimen handling. The second part comprised information about haematology specimen acceptability. In this part, participants were asked to register all specimens submitted to the laboratory for haematology testing which were determined to be rejected from 1^st^ to 31^th^ July. For each rejected specimen, the following information had to be recorded: source department, container type, container material type, transportation method, phlebotomy personnel, reason for rejection, action taken and time (including time of specimen receipt, time of specimen rejection and time of specimen recollected or relabelled). The above information was selected from a drop menu. Furthermore, participants also provided the total number of haematology specimens (both acceptable for testing and rejected) submitted in the same time period and the total number of specimens by source department, container type, container material type, transportation method and phlebotomy personnel.

Laboratories which provided questionnaires without the total number of specimens submitted or with incomplete data were excluded. Furthermore, laboratories which registered not only haematology specimens but also specimens of other branches (such as chemistry or immunology), were not included in the analysis.

### Statistical analysis

Excel (Microsoft, Redmond, WA) (2007 version) and SPSS 20.0 (IBM SPSS Inc., Chicago, USA) were used to analyse collected data. The overall rejection rate and interlaboratory rejection rate were calculated by the number of rejected specimens *per* total specimens. Percentage (%) and sigma value (σ) were employed to evaluate the status of rejection rate and data were presented as percentiles (p5, p25, p50, p75, p95) of distribution. The characteristics and practices of institutions were considered and participants were grouped according to them. The data of submitted and rejected specimens were aggregated and the Odds ratio (OR) was calculated, for each level, by dividing the percentage of rejected specimen by the baseline percentage of rejected specimen. The OR and their associated 95% confidence intervals (CI) were calculated, and it was considered to be statistically significant if the associated 95% CI didn’t include the value 1.00. Besides, specimen processing delay, defined as the interval between the original and recollected/relabelled specimen receipt time, was calculated.

Shapiro-Wilks (N ≤ 50) and Kolmogorov-Smirnov modified by Lilliefors (N>50) were used to test the normality of the data. Nonparametric Kruskal-Wallis test for three or more groups and the Mann-Whitney U test for two groups were performed to compare the interlaboratory rejection rate by features of institutions and the specimen processing delay in different departments. Significance was defined as probability, P-value<0.05.

## Results

A total of 1586 questionnaires were sent, among which, 890 questionnaires were returned, giving a response rate of 56% (890/1586). According to exclusion criteria, 580 questionnaires were considered valid and included in further analysis. Out of these, tertiary hospital accounted for 77%, with only 1.2% were accredited by CAP and 11% by ISO 15189. Most of participants (69%) were general hospitals and 15% were specialized hospitals (Table 1).

**Table 1:** Questions and answers delivered to participants

**Questions and possible answers**	**Number of laboratories, N (%)**
**1. What grade does your hospital belong to?**	
A. Class III Grade I	342 (59.0)
B. Class III Grade II	103 (17.7)
C. Under Class II	135 (23.3)
**2. What category does your hospital belong to?**	
A. General hospital	400 (69.0)
B. Specialized hospital	87 (15.0)
C. Traditional Chinese and western medicine hospital	16 (2.8)
D. Traditional Chinese medicine hospital	46 (7.9)
E. Maternal and child-care service centers	26 (4.5)
F. Others	5 (0.8)
**3. How many beds does your laboratory occupy?**	
A. 0 - 500	136 (23.4)
B. 501 - 1000	204 (35.2)
C. 1001 - 1500	128 (22.1)
D. more than 1500	112 (19.3)
**4. Is your laboratory accredited by CAP?**	
A. Yes	7 (1.2)
B. No	573 (98.8)
**5. Is your laboratory accredited by ISO 15189?**	
A. Yes	64 (11.0)
B. No	516 (89.0)
**6. What is the most common phlebotomy personnel in inpatient department of your hospital?**	
A. Nurse (managed by nursing department)	560 (96.5)
B. Nurse (managed by clinical laboratory)	9 (1.6)
C. Laboratory personnel	3 (0.5)
D. Clinician	3 (0.5)
E. Others	5 (0.9)
**7. What is the most common phlebotomy personnel in outpatient department of your hospital?**	
A. Nurse (managed by nursing department)	227 (39.1)
B. Nurse (managed by clinical laboratory)	149 (25.7)
C. Laboratory personnel	195 (33.6)
D. Others	9 (1.6)
**8. What is the most common phlebotomy personnel in emergency department of your hospital?**	
A. Nurse (managed by nursing department)	418 (72.1)
B. Nurse (managed by clinical laboratory)	38 (6.6)
C. Laboratory personnel	111 (19.1)
D. Others	13 (2.2)
**9. What is the most common way of specimen transportation for inpatient department of your hospital?**	
A. Laboratory personnel	27 (4.7)
B. Pneumatic tube system	31 (5.3)
C. Full-time transportation worker	390 (67.2)
D. Nurse	71 (12.2)
E. Patient or relation	4 (0.7)
F. Company	48 (8.3)
G. Others	9 (1.6)
**10. What is the most common way of specimen transportation in outpatient department of your hospital?**	
A. Laboratory personnel	256 (44.2)
B. Pneumatic tube system	17 (2.9)
C. Full-time transportation worker	188 (32.4)
D. Nurse	65 (11.2)
E. Patient or relation	25 (4.3)
F. Company	23 (4.0)
G. Others	6 (1.0)
**11. What is the most common way of specimen transportation in emergency department of your hospital?**	
A. Laboratory personnel	44 (7.6)
B. Pneumatic tube system	23 (4.0)
C. Full-time transportation worker	261 (45.0)
D. Nurse	125 (21.6)
E. Patient or relation	86 (14.8)
F. Company	31 (5.3)
G. Others	10 (1.7)
**12. What’s the training frequency for venous blood collection in your hospital?**	
A. Once a year	218 (37.6)
B. Once halt a year	94 (16.2)
C. Once every 1 to 3 months	50 (8.6)
D. Irregularly	202 (34.8)
E. Others	16 (2.8)
**13. Does your laboratory establish a specimen rejection criterion?**	
A. Yes	571 (98.4)
B. No	9 (1.6)
**14. Does your laboratory routinely review main reasons for specimen rejection and take measures on it?**	
A. Yes	505 (87.1)
B. No	75 (12.9)
**15. Does your laboratory monitor time from phlebotomy to specimen admission for tubes sampled outside of laboratory?**	
A. Yes	369 (63.6)
B. No	211 (36.4)
**16. Does your laboratory monitor temperature conditions for tubes sampled outside of laboratory?**	
A. Yes	112 (19.3)
B. No	468 (80.7)
**17. How does your laboratory record the rejected specimens?**	
A. Record on paper	263 (45.3)
B. Record in the computer system	141 (24.3)
C. Both record on paper and in the computer system	165 (28.5)
D. Do not record	3 (0.5)
E. Other	8 (1.4)
**18. What actions does your laboratory take when reporting a rejected specimen?**	
A. Explain the reason why the specimen is rejected and returned	24 (4.1)
B. Explain the reason why the specimen is rejected and returned, and request to recollect	242 (41.7)
C. Do not return the specimen, but explain the reason why the specimen is rejected	5 (0.9)
D. Do not return the specimen, but explain the reason why the specimen is rejected, and request to recollect	306 (52.8)
E. Other	3 (0.5)
ISO - International Organization for Standardization. CAP - College of American Pathologists.	

Nurse managed by nursing department was the most frequent phlebotomy personnel in inpatient department (97%) and emergency department (72%). As for outpatient department, the common phlebotomy personnel were nurse managed by nursing department (39%), laboratory personnel (34%) and nurse managed by clinical laboratory (26%). Common specimen transportation methods were by full-time transportation worker (67%) and nurse (12%) in inpatient department; full-time transportation worker (32%) and laboratory personnel (44%) in outpatient department; full-time transportation worker (45%) and nurse (22%) in emergency department. The proportion of participants using a pneumatic tube system for transportation in the three departments were 5%, 3% and 4%, respectively. Table 1 summarizes all the responses received from participants in the questionnaire.

**Figure 1:**
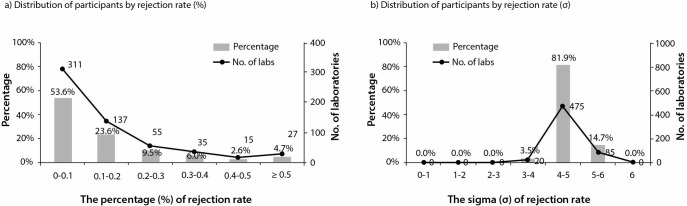
Distribution of participants according to rejection rate (% and σ) of haematology specimens

Out of 10,181,036 reported haematology specimens submitted to the participating laboratories, 11,447 (0.11%) were rejected, and the σ of the overall rejection rate was 4.6. The distribution of percentiles of the rejection rate (% and σ) were as follows: the 5^th^, 25^th^, 50^th^, 75^th^ and 95^th^ for percentage was 0.01%, 0.04%, 0.09%, 0.18% and 0.50%, and for σ scale was 4.1, 4.4, 4.6, 4.9 and 5.2, respectively. Figure 1 shows the distribution of participating laboratories according to the percentage and σ scale of haematology specimens rejected. A total of 311, 137 and 55 laboratories, respectively, provided a rejection rate lower than 0.1%, between 0.1% and 0.2%, and between 0.2% and 0.3%. Most of laboratories (82%) had a σ between 4σ and 5σ.

Table 2 illustrates the specimen rejection rate of participants according to their characteristics/practices, including hospital grade, bed size, ISO 15189/CAP accreditation, training frequency for phlebotomy personnel, specimen rejection criteria, and periodically reviewed main reasons for rejection and take measures. No significant differences were identified.

**Table 2:** Specimen rejection rate by institution characteristics/practices

**Institution characteristics/practices**		**N**	**Median of specimen rejection rate, %**	**P**
**Hospital grade**	Class III Grade I	342	0.10	0.200
	Class III Grade II	103	0.07	
	Under Class II	135	0.08	
**Bed size**	0 - 500	136	0.10	0.508
	501 - 1000	204	0.09	
	1001 - 1500	128	0.09	
	> 1500	112	0.08	
**ISO 15189/CAP accreditation**	Yes	66	0.12	0.065
	No	514	0.09	
**Training frequency**	Once a year	218	0.10	0.513
	Once half a year	94	0.08	
	Once every one to three months	50	0.09	
	Irregularly scheduled	202	0.09	
**Rejection criteria**	Yes	571	0.09	0.157
	No	9	0.04	
**Periodical summary of specimen rejection reasons**	Yes	505	0.09	0.608
	No	75	0.09	
Training frequency - training frequency for phlebotomy personnel (question 12 in Table 1). Rejection criteria - refer to question 13 in in Table 1. Periodical rejection summary - refers to the periodical review of main reasons for specimen rejection (question 14 in Table 1). ISO - International Organization for Standardization. CAP - College of American Pathologists. Data were analysed using Kruskal-and Mann-Whitney U test. P-value < 0.05 was considered statistically significant.				

Table 3 compares the percentage of collected and rejected specimens according to specimen characteristics, including source department, container type, container material type, transportation method and phlebotomy personnel. Odds ratio and 95% CI limits are presented in the last column in Table 3.

**Table 3:** Relation between specimen characteristics and specimen collection/rejection

**Specimen characteristic**	**Total specimens, N**	**Rejected specimens, N (%)**	**Rejection rate, %**	**OR (95% CI)**
**Department**				
Inpatient	5,155,339	9983 (87.2)	0.19	baseline
Outpatient	3,894,495	761 (6.7)	0.02	0.10 (0.09 - 0.11)
Emergency	1,131,202	703 (6.1)	0.06	0.32 (0.30 - 0.35)
**Container type**				
Vacuum tube	10,114,926	11,330 (99.0)	0.11	baseline
Non-vacuum tube (syringe)	66,110	117 (1.0)	0.18	1.16 (1.32 - 1.90)
**Container material type**				
Plastic	7,329,735	7944 (69.4)	0.11	baseline
Glass	2,851,301	3503 (30.6)	0.12	1.13 (1.09 - 1.18)
**Transportation method**				
Full-time worker	6,245,398	8843 (77.3)	0.14	baseline
Pneumatic tube system	761,618	959 (8.4)	0.13	0.89 (0.83 - 0.95)
Nurse	856,992	906 (7.9)	0.11	0.75 (0.70 - 0.80)
Laboratory personnel	2,040,421	539 (4.7)	0.03	0.19 (0.17 - 0.20)
Patient or relation	220,408	175 (1.5)	0.08	0.56 (0.48 - 0.65)
Other	56,199	25 (0.2)	0.04	-
**Phlebotomy personnel**				
Nurse A	7,354,885	10,979 (95.9)	0.15	baseline
Nurse B	1,329,202	263 (2.3)	0.02	0.13 (0.12 - 0.15)
Laboratory personnel	1,466,568	139 (1.2)	0.01	0.06 (0.05 - 0.08)
Clinician	13,406	40 (0.4)	0.30	2.00 (1.47 - 2.73)
Other	16,975	26 (0.2)	0.15	-
Nurse A - managed by nursing department. Nurse B - managed by clinical laboratory. OR - odds ratio. CI - confidence interval. OR was calculated by dividing the percentage of rejected specimen by the baseline of rejected specimen.				

When compared with source department, 87% of rejection specimens were from inpatient department, with a rejection rate of 0.19%, which was significantly higher than emergency department. The OR demonstrated that non-vacuum tube had a significantly greater rejection rate than vacuum tube. The OR between plastic and glass was 1.13, which showed statistical significance, but this appeared to be of little practical significance. Taking transportation method into account, transportation by a full-time transportation worker had a slightly higher rejection rate than other methods, among which, specimens transported by laboratory personnel showed an apparently lower rejection rate, where the OR was 0.19. As for phlebotomy personnel, the OR revealed a significantly lower rate of rejection for specimens drawn by laboratory personnel (0.06) and nurse managed by clinical laboratory (0.13) when compared to nurse managed by nursing department, and specimens drawn by clinicians had a little higher rejection rate than that of nursing staff.

Table 4 illustrates the distribution of rejected specimens in this study based on reasons for rejection. In total, about 85% of rejections attributed to the five reasons: specimen clotted (57%), insufficient specimen volume (14%), inappropriate specimen-anticoagulant volume ratio (6.9%), incorrect container type (4.2%) and inadequately labelled (2.9%).

**Table 4:** Reasons for haematology specimen rejection

**Reason for rejection**	**Number of specimens, N (%)**
Specimen clotted	6548 (57.2)
Insufficient specimen volume	1602 (14.0)
Inappropriate specimen-anticoagulant volume ratio	791 (6.9)
Incorrect container type	476 (4.2)
Inadequately labelled	326 (2.9)
Specimen haemolysed	306 (2.7)
Lipemia	262 (2.3)
Empty tube	177 (1.6)
Incorrect specimen type	144 (1.3)
Inappropriate time in specimen collection	133 (1.2)
Inappropriate test request	121 (1.1)
Payment related	104 (0.9)
Medical orders modified	94 (0.8)
Excessive transportation time	60 (0.5)
Specimen collected on the same side of infusion	47 (0.4)
Specimen not received	19 (0.2)
Testing parameters beyond the scope of haematology laboratory	18 (0.2)
Inappropriate transportation condition	7 (0.1)
Other reasons	212 (1.9)

Of the 11,447 rejected specimens, laboratory staff were unable to obtain information about measures taken of 562 (4.9%) specimens, requested 9563 (83.6%) specimens to be recollected, and asked for 479 (4.2%) specimens to be relabelled, and 2.6% rejected specimens were abandoned by the laboratory and provider. Besides, 541 (4.7%) rejection were classified as “other”, which participants did not believe fit into one of the provided actions.

Table 5 shows that the median specimen processing delay was statistically significant among different source department. The overall median specimen processing delay due to specimen rejection was 57.0 minutes for outpatient specimens, 43.3 minutes for emergency specimens and 81.0 minutes for inpatient specimens. The result indicated that the median specimen processing delay in inpatient department was greater than which in outpatient department (P < 0.001) and emergency department (P < 0.001), whereas there was no significance between outpatient department and emergency department (P = 0.111).

**Table 5:** Median specimen processing delay in the three departments investigated

**Indicator**	**Source department**	**N**	**P_5_**	**P_25_**	**P_50_**	**P_75_**	**P_95_**	**P**
**Median specimen** **processing** **delay, min**	Outpatient	177	9.0	25.0	57.0	106.3	1273.2	< 0.001
	Emergency	136	10.0	25.0	43.3	80.3	191.8	
	Inpatient	430	21.8	49.4	81.0	125.6	977.3	
P_5_ - 5^th^ percentile. P_25_ - 25^th^ percentile. P_50_ - 50^th^ percentile. P_75_ - 75^th^ percentile. P_95_ - 95^th^ percentile. Kruskal-Wallis test was used for data analysis. P ≤ 0.05 was considered statistically significant.								

## Discussion

The rejection rate in the study was similar to, but slightly lower than that found in the previous complete blood count testing study in 2012 (*11*). The comparison, to some extent, illustrates that laboratories in China pay more attention to collection and reception of specimens in the past 5 years, leading to a reduction in rejection due to preanalytical errors. However, it is worth mentioning that the apparently good sigma values in the study should not mislead laboratories and oversight bodies to believe that there were no issues to address in preanalytical quality.

Compared with the survey conducted by CAP and in Spain, the overall rejection rate in this study was much lower, which might be attributed to the following reasons (*3*, *5*, *7*). First, participants in the survey were mainly tertiary hospitals, which represents a relatively good laboratory practice in China. Second, LIS in some laboratories had no adequate function to record all the information of specimens. A lot of participants responded that they recorded rejected specimens manually and some of them believed that not all the rejected specimens were recorded. Third, the definition of unacceptable specimen varied among different laboratories and laboratories had different rejection criteria. That means, the same type specimen might be rejected by the laboratory but might not be rejected by the other laboratory.

The most common rejection reason was clotted specimen, which occurred four times more often than the second most common reason for rejection, insufficient specimen volume. The result was similar to the study of haematology specimen acceptability conducted by CAP, whose most common reasons for rejection was clotted and insufficient specimen quantity (*5*). Since the predominance of clotted specimens, participants were suggested to have an in-depth investigation for reasons for that. For example, the blood collection procedure and the mixing procedure of collection tube, so as to decrease the occurrence of clotted specimen.

It is worth mentioning that transportation method and phlebotomy personnel were important influence factors for specimen acceptability. Specimens transported by laboratory personnel and specimens collected by laboratory personnel and nurse managed by laboratory exhibited a much lower rejection rate. It possibly because laboratory personnel had a better knowledge of the influence factors of specimen and had a better practice on specimen collection and transportation. The results mean that participants might benefit from strengthening training for phlebotomy and transportation personnel.

The common direct consequences of specimen rejection were the need to recollect a new specimen and result in delay in the examination and reporting of the specimen. The outcomes of specimen recollection and delay in reporting usually led to patient complaint and dissatisfaction and postponed the diagnosis and treatment for the patient.

A survey on specimen acceptability in China was conducted in 2012 (*11*). However, considering it was an initial investigation on quality indicators conducted by NCCL in China, the content of the investigation and the data returned was not complete. Compared to that, this study had a detailed investigation on the practice of the management in preanalytical phase and had a detailed analysis on the relation between specimen acceptability and specimen characteristics and quantified the consequence of specimen processing delay. However, there was certain limitation in the study. Participants were asked to register all the information of rejected specimens, one by one, which was a little complicated and hard to complete.

In summary, the management of unacceptable specimens in China needs to be improved. The criteria for specimen rejection and judgment method for unacceptable specimens need to be harmonized. Specimen rejection rate was an important indicator for quality monitoring in preanalytical phase. Laboratories themselves should regularly review the monthly specimen rejection rate and related influence factor and take effective actions. Strengthening information system construction played a key role in the process. The main objective of EQA program for specimen acceptability was to provide a useful tool for laboratories to better follow up the status and achieved the quality continuous improvement. The platform and framework for specimen acceptability in the study would be helpful for long-term monitoring of quality indicators for clinical laboratories, but the questionnaire in this study was a little complicated. In order to have a more convenient and correct fill in of answer forms, simplification and modification should be introduced in the following investigation.
